# Radiomics-Based Preoperative Assessment of Muscle-Invasive Bladder Cancer Using Combined T2 and ADC MRI: A Multicohort Validation Study

**DOI:** 10.3390/jimaging11100342

**Published:** 2025-10-01

**Authors:** Dmitry Kabanov, Natalia Rubtsova, Aleksandra Golbits, Andrey Kaprin, Valentin Sinitsyn, Mikhail Potievskiy

**Affiliations:** 1 Department of Computed Tomography and Magnetic Resonance Imaging, P. Hertsen Moscow Oncology Research Institute (MORI), 125284 Moscow, Russia; 2 Department of Computed Tomography and Magnetic Resonance Imaging, N. Lopatkin Scientific Research Institute of Urology and Interventional Radiology (SRIUIR), 105425 Moscow, Russia; 3 Department of Oncology and Radiology, Institute of Medicine, Peoples’ Friendship University of Russia—RUDN University, 117198 Moscow, Russia; 4 Radiology Department of University Medical Center, Lomonosov Moscow State University, 119991 Moscow, Russia; 5 Center for Clinical Trials of Center for Innovative Radiological and Regenerative Technologies, Federal State Budgetary Institution National Medical Research Radiological Centre of the Ministry of Health of the Russian Federation, 249031 Obninsk, Russia

**Keywords:** radiomics, bladder cancer, MRI, texture analysis, muscle invasion, staging, MIBC, LifeX

## Abstract

Accurate preoperative staging of bladder cancer on MRI remains challenging because visual reads vary across observers. We investigated a multiparametric MRI (mpMRI) radiomics approach to predict muscle invasion (≥T2) and prospectively tested it on a validation cohort. Eighty-four patients with urothelial carcinoma underwent 1.5-T mpMRI per VI-RADS (T2-weighted imaging and DWI-derived ADC maps). Two blinded radiologists performed 3D tumor segmentation; 37 features per sequence were extracted (LifeX) using absolute resampling. In the training cohort (*n* = 40), features that differed between non-muscle-invasive and muscle-invasive tumors (Mann–Whitney *p* < 0.05) underwent ROC analysis with cut-offs defined by the Youden index. A compact descriptor combining GLRLM-LRLGE from T2 and GLRLM-SRLGE from ADC was then fixed and applied without re-selection to a prospective validation cohort (*n* = 44). Histopathology within 6 weeks—TURBT or cystectomy—served as the reference. Eleven T2-based and fifteen ADC-based features pointed to invasion; DWI texture features were not informative. The descriptor yielded AUCs of 0.934 (training) and 0.871 (validation) with 85.7% sensitivity and 96.2% specificity in validation. Collectively, these findings indicate that combined T2/ADC radiomics can provide high diagnostic accuracy and may serve as a useful decision support tool, after multicenter, multi-vendor validation.

## 1. Introduction

Bladder cancer (BC) is the most common malignancy of the urinary tract. Prognosis and management primarily depend on the depth of invasion: muscle involvement (stage ≥ T2) is associated with poorer survival and necessitates more aggressive treatment [1,2]. Current guidelines recommend transurethral resection of bladder tumor (TURBT) with histopathologic evaluation to distinguish non-muscle-invasive (NMIBC) from muscle-invasive bladder cancer (MIBC) and to determine tumor stage and aggressiveness [3,4]. However, TURBT is invasive and operator-dependent, complications (hematuria, urinary tract infection, bladder perforation) may occur, and under-staging is reported in up to 25% of cases [5,6,7]. Accordingly, there is a clear need for accurate noninvasive preoperative assessment of invasion depth; quantitative, reproducible imaging biomarkers that complement visual interpretation may help address these limitations.

Multiparametric magnetic resonance imaging (mpMRI) has shown promise for assessing muscle invasion [8]. The implementation of standardized reporting systems has improved diagnostic accuracy and reduced interobserver variability, yet a substantial learning curve persists, with lower concordance among less experienced readers [9,10]. Beyond primary staging, extensions of the VI-RADS framework have been proposed for treatment response assessment; for example, neoadjuvant chemotherapy VI-RADS (nacVI-RADS) has demonstrated good accuracy and inter-reader agreement for post-therapy evaluation, underscoring the value of standardized mpMRI scoring [11]. However, VI-RADS remains a qualitative, reader-dependent score: equivocal categories (e.g., VI-RADS 3–4) and site-to-site variability can limit consistency, particularly early in the learning curve, and visual criteria may underutilize voxel-level heterogeneity captured by mpMRI.

Radiomics—an imaging science that combines medical imaging, quantitative feature extraction, and machine learning—offers tools for disease detection, staging, prognosis, and treatment-response prediction [12]. Among its core methods, texture analysis quantitatively characterizes gray-level distributions and spatial relationships (e.g., GLCM, GLRLM) within a region of interest, providing objective measures of intratumoral heterogeneity that are not apparent on visual inspection. By converting segmented images into reproducible numerical descriptors, radiomics may help reduce observer dependence and reveal pathophysiologic patterns [13,14]. In this sense, radiomics is not an added layer of complexity but a complementary, quantitative framework that can standardize risk estimation across readers and centers, assist decision-making in borderline cases, and be integrated alongside VI-RADS without additional scan time. To further minimize workflow burden, we operationalized radiomics as a compact, interpretable two-feature descriptor derived from routine T2-weighted and ADC data.

Here, we aimed to develop and validate an mpMRI-based radiomics approach for preoperative identification of muscle invasion (≥T2) in bladder cancer.

## 2. Materials and Methods

### 2.1. Study Design

This study was structured as a sequential, two-cohort investigation. Initially, a retrospective training cohort was formed, in which all extracted radiomic texture features and potential descriptors were systematically analyzed to identify those most strongly associated with muscle invasion in bladder cancer. Based on the results of this analysis, a radiomic descriptor was developed.

After this, an independent prospective validation cohort was assembled. The effectiveness and diagnostic accuracy of the developed descriptor were then tested on this cohort using a focused analytical pipeline: only the previously selected radiomic features and descriptor were applied, without repeating the comprehensive feature selection performed in the training group.

The main steps of this study included

- Patient selection and assignment to retrospective (training) cohort;
- Multiparametric MRI examination;
- Manual tumor segmentation by radiologists;
- Extraction and analysis of radiomic texture features in the training cohort;
- Development of a radiomic descriptor associated with muscle invasion;
- Prospective recruitment of the validation cohort;
- Application and validation of the descriptor in the independent cohort;
- Statistical assessment of diagnostic performance.

**Figure 1:**
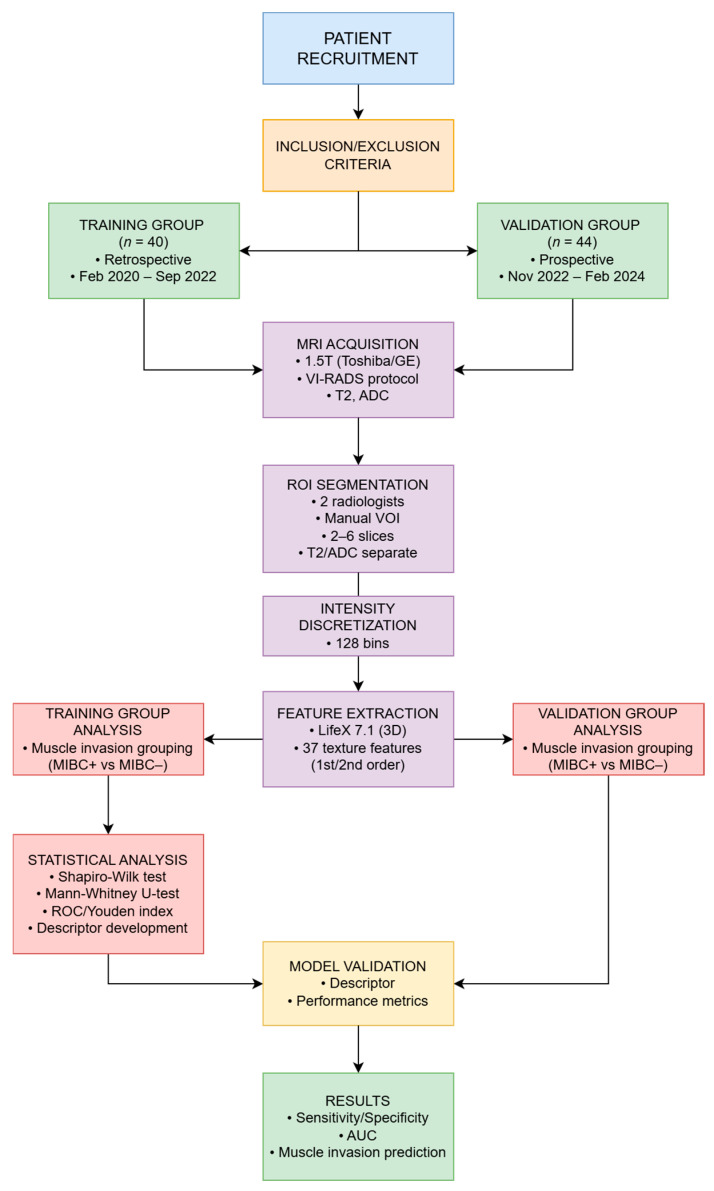
Overall workflow of the study.

Features were screened exclusively in the retrospective training cohort; the fixed two-feature descriptor was then applied to the prospective validation cohort without re-selection or re-tuning. This design emulates real-world deployment while mitigating data leakage. The sequence of study stages is summarized in Figure 1.

### 2.2. Population Structure

Two patient cohorts were recruited: a retrospective training cohort (*n* = 40) and a prospective validation cohort (*n* = 44). The training cohort included patients who underwent preoperative MRI at the Department of Urology, P. Hertsen Moscow Oncology Research Institute, between February 2020 and September 2022. Eligible patients of both sexes had histologically confirmed bladder cancer based on specimens obtained within 6 weeks after MRI (via TUR, TUR-biopsy, or cystectomy).

The prospective validation cohort included patients enrolled between November 2022 and February 2024, with histopathological data obtained at least 6 weeks post-MRI.

The training and validation time windows did not overlap. The two-feature descriptor defined in the training cohort was locked and applied unchanged to the prospective validation cohort—without re-selection, re-tuning, or threshold adjustment—to emulate clinical deployment and minimize information leakage; diagnostic performance (AUC, sensitivity, specificity) is reported with 95% confidence intervals.

Histopathological examination served as the reference standard for muscle invasion (≥T2 stage) in both cohorts. Histopathology obtained after TURBT or radical cystectomy, when performed for clinical indications, served as the reference standard within ≤6 weeks of MRI. When both TURBT and cystectomy histology were available, the cystectomy result defined the reference for muscle invasion. Muscularis propria was present in all TURBT specimens (100%).

Inclusion criteria for both cohorts included (1) a tumor mass visible on MRI, (2) informed consent, and (3) histopathology from TURBT with muscularis propria present or cystectomy, obtained within ≤6 weeks of MRI. Exclusion criteria included (a) absolute contraindications for MRI (e.g., cardiac pacemaker, defibrillator, metallic implants), (b) TUR and/or intravesical chemotherapy or BCG therapy within 2 weeks before MRI, (c) MRI performed less than 3 days after Foley catheter removal or cystoscopy, and (d) empty bladder during imaging.

Patients in the training cohort were stratified based on the presence or absence of muscle invasion, based on histopathology. Descriptive statistics were calculated for all texture parameters, optimal cut-off points were determined using the Youden index, and ROC curves were constructed. Differences were analyzed using the Mann–Whitney U test and Pearson’s chi-square test. The overall analysis workflow is illustrated in Figure 1.

The validation cohort was analyzed using the same approach: patients were divided into two subgroups according to histopathology, followed by texture analysis. The radiomic signature developed in the training cohort was then applied to the validation cohort.

Patients with histologically confirmed bladder cancer were divided into retrospective (training) and prospective (validation) cohorts according to the inclusion and exclusion criteria. All patients underwent 1.5 T MRI with the VI-RADS protocol. Tumors were segmented by two radiologists, and texture features were extracted from T2-weighted and DWI/ADC images after signal intensity discretization. In the training cohort, features were analyzed for association with muscle invasion, and a multiparametric radiomic descriptor was developed. This descriptor was then validated in the prospective cohort. Statistical analyses included the Shapiro–Wilk test, Mann–Whitney U-test, and ROC curve analysis. The workflow resulted in a validated radiomic model for predicting muscle invasion in bladder cancer.

### 2.3. MRI Data Acquisition

All MR examinations were performed using high-field 1.5 T scanners (Toshiba Vantage Titan 1.5 T (Toshiba Medical Systems Corporation, Otawara, Japan) and GE SIGNA Voyager 1.5 T (GE Healthcare, Waukesha, WI, USA)) equipped with multichannel abdominal phased array coils. Imaging protocols followed the VI-RADS system to ensure reproducibility across scanners [9]. The scanner model was recorded, and potential multi-vendor effects are acknowledged in the Section 4.4. The MRI protocol included T2-weighted imaging in two planes, and diffusion-weighted imaging (DWI) in axial and sagittal planes, with a slice thickness of 4 mm. DWI was performed using a single-shot, spin-echo echo-planar imaging sequence (b-values: 0 and 800 s/mm^2^). Apparent diffusion coefficient (ADC) maps were calculated voxel by voxel using a monoexponential model.

### 2.4. ROI Extraction

All MR images were independently analyzed by two radiologists (with 8 and 10 years of experience in pelvic imaging), both blinded to histopathology. Disagreements were resolved via consensus.

**Figure 2:**
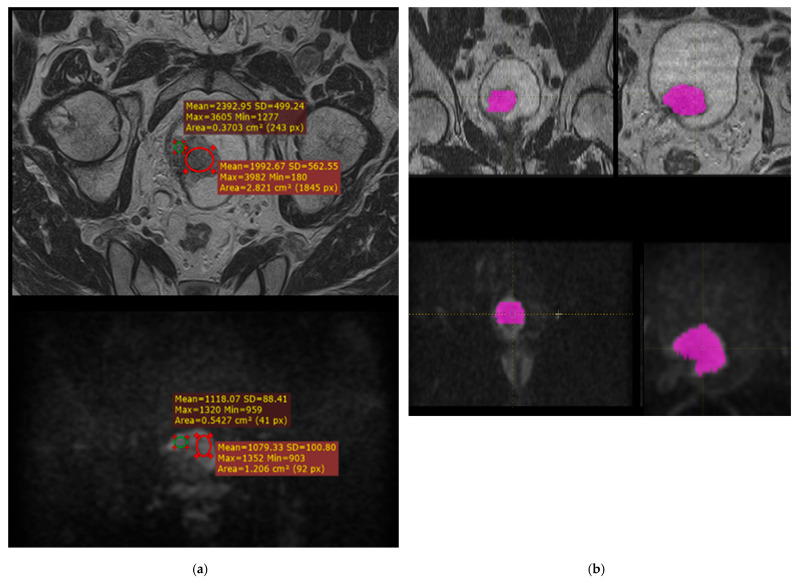
Radiomics workflow for preoperative MRI assessment of bladder cancer. (**a**) Example of region of interest (ROI) placement on T2-weighted (**top**) and diffusion-weighted (**bottom**) MR images for determination of minimum and maximum signal intensities. These measurements were used to select the bin size for absolute presampling during texture feature extraction. (**b**) Manual segmentation of the tumor volume of interest (VOI) on T2-weighted (**top**) and diffusion-weighted (**bottom**) MRI sequences. The VOI was delineated separately on each sequence by two radiologists for subsequent radiomic texture analysis performed using LifeX 7.1 software.

For small tumors, the volume of interest (VOI) was manually delineated on 2–3 slices, and for larger tumors, on up to 5–6 slices. Due to variable slice positioning (caused by bladder filling, motion, or angulation), VOIs were assigned separately for T2-weighted and DWI sequences (Figure 2). Tumor margins were delineated more accurately on T2-weighted images due to the high signal intensity of urine. For ADC maps, the same VOI as for DWI was used.

Only axial slices were used for analysis in all sequences. In cases of multifocal disease, the lesion with the largest axial diameter and/or deepest invasion was selected. Slices for texture analysis were selected similarly, using the lesion with the greatest depth of invasion and/or largest dimensions.

### 2.5. Feature Extraction

For texture analysis, absolute presampling (fixed-bin discretization) was used, as recommended for MRI. Minimum and maximum signal intensities for each sequence were measured on representative tumor slices using Radiant DICOM Viewer 2.3 (at least two measurements for tumors < 10 mm and at least five for tumors ≥ 10 mm) to determine the scaling for absolute presampling. For T2-weighted images, the minimum and maximum intensities were 175 and 3152, respectively; for DWI, 50 and 5300; and for ADC, 20 and 2550. The number of gray levels was fixed at 128 for all sequences, in line with the LifeX developer’s guidance for MRI and widely adopted practice in MRI radiomics, which balances quantization noise and feature stability. The same discretization parameters (absolute presampling and 128 gray levels) and intensity ranges were applied identically in the training and prospective validation cohorts to ensure comparability.

Texture analysis was performed with LifeX 7.1 (www.lifexsoft.org) in three dimensions (3D), on T2, DWI, and ADC sequences. Thirty-seven features were extracted from each sequence, including first- and second-order statistics: intensity histograms (6 features), a gray-level co-occurrence matrix (GLCM), a gray-level run-length matrix (GLRLM, 11 features), a neighborhood gray-level difference matrix (NGLDM, 3 features), and a gray-level zone length matrix (GLZLM, 11 features) (see Table 1).

### 2.6. Software

The study database was created and maintained using Microsoft Excel 2016 (Microsoft, Redmond, WA, USA). Statistical analyses were performed using Statistica Professional 12 (StatSoft, Tulsa, OK, USA) and Microsoft Excel 2016. Radiant DICOM Viewer 2.3 (Medixant, Poznań, Poland) and built-in tools of LifeX 7.1 (Institut Curie Centre de Recherche, Paris, France) were utilized for the viewing and analysis of DICOM medical images.

### 2.7. Statistical Analysis

Descriptive statistics were calculated for demographic, clinical, and texture features. For quantitative variables, results are presented as the mean, median, standard deviation (SD), minimum, maximum, and the IQR. For qualitative variables, absolute and relative frequencies are reported.

The normality of the data distribution was assessed using the Shapiro–Wilk test. All variables, except patient age, were not normally distributed. Intergroup comparisons were performed using the Mann–Whitney U-test for quantitative variables and Pearson’s chi-square test for categorical variables.

Diagnostic performance was assessed via receiver operating characteristic (ROC) curve analysis, with the calculation of the area under the curve (AUC). ROC analysis was performed for each parameter relative to the histopathological reference standard (muscle invasion status). The optimal cut-off value for each feature was determined using the Youden index. A *p*-value < 0.05 was considered statistically significant. Results are reported as median ± standard deviation (MD ± SD), unless otherwise specified.

Overfitting control. All feature screenings were performed exclusively in the training cohort. The final two-feature descriptor was then fixed (locked) based on training results, and the same thresholds were applied to the validation cohort without re-tuning or re-selection. Diagnostic accuracy was summarized by ROC AUC with 95% confidence intervals, and an achieved power/precision analysis was reported for the primary endpoint.

Multiplicity control. Feature screening in training was exploratory and intended solely to derive a compact model; we did not conduct multiplicity-adjusted hypothesis testing for individual features. Instead, multiplicity was controlled at the model level by restricting the final signature to two features and evaluating it on a temporally independent validation cohort.

### 2.8. Sample Size and Power

Achieved power for the primary endpoint (ROC AUC vs. 0.5, two-sided α = 0.05) was computed using the Hanley–McNeil variance and the actual case/control counts. For the training cohort (*n* = 40; MIBC/NMIBC 21/19; AUC = 0.934), the standard error was 0.041 (95% CI 0.853–1.000) with power ≈ 1.00. For the prospective internal validation cohort (*n* = 44; 28/16; AUC = 0.871), the standard error was 0.053 (95% CI 0.768–0.974) with power ≈ 1.00. With the same sample sizes, the minimally detectable AUC at 80% power is ~0.72.

## 3. Results

A total of 40 retrospective patients (training cohort) and 44 prospective patients (validation cohort) were included in this study. The presence of muscle invasion was determined via postoperative histopathological assessment (TURBT or cystectomy). There were no significant differences between the cohorts in terms of sex, age, or tumor characteristics (muscle invasion status, T stage). Muscularis propria was present in all TURBT specimens (100%). When both TURBT and cystectomy histology were available, the cystectomy result defined the reference for muscle invasion. Detailed patient characteristics are summarized in Table 2.

Within the training cohort, patients were stratified into two subgroups based on the presence or absence of muscle invasion according to postoperative histology. Individual texture features from each patient were compared between subgroups using the Mann–Whitney U-test. For each MRI sequence (T2, DWI, and ADC), 37 texture features were analyzed separately.

Statistically significant differences between subgroups were identified for 11 T2-derived features and 15 ADC-derived features; no significant differences were observed among DWI-derived features. The most frequently discriminative features were derived from gray-level run length matrices (GLRLMs) and gray-level zone length matrices (GLZLMs). For each significant feature, receiver operating characteristic (ROC) curve analysis with cut-off point determination was performed using the Youden index, yielding relatively high sensitivity and specificity (75–95%) and an area under the curve (AUC) ranging from 0.701 to 0.914. The features showing significant intergroup differences (by Mann–Whitney U-test) are listed in Table 3.

Although many radiomic features showed significant subgroup differences, using a large set of parameters is impractical for clinical deployment and increases the risk of overfitting in modest samples. To facilitate application, we predefined a compact two-feature descriptor integrating the top univariate performers from the training cohort: GLRLM-LRLGE from T2-weighted images and GLRLM-SRLGE from ADC maps. These features consistently achieved among the highest AUC values with balanced sensitivity/specificity and provide complementary information—T2 reflects morphologic contrast, whereas ADC captures diffusion-related microstructural heterogeneity—thereby avoiding same-sequence redundancy while keeping the model interpretable. For each feature, the optimal cut-off was determined with the Youden index in training and fixed at 3731.71 (T2-LRLGE) and 3785.45 (ADC-SRLGE) for use in validation without re-selection or re-tuning.

The combined descriptor was defined as positive for muscle invasion if both individual feature values exceeded their respective cut-off thresholds. This approach was chosen to maximize specificity while maintaining high sensitivity for muscle-invasive disease detection.

**Figure 3:**
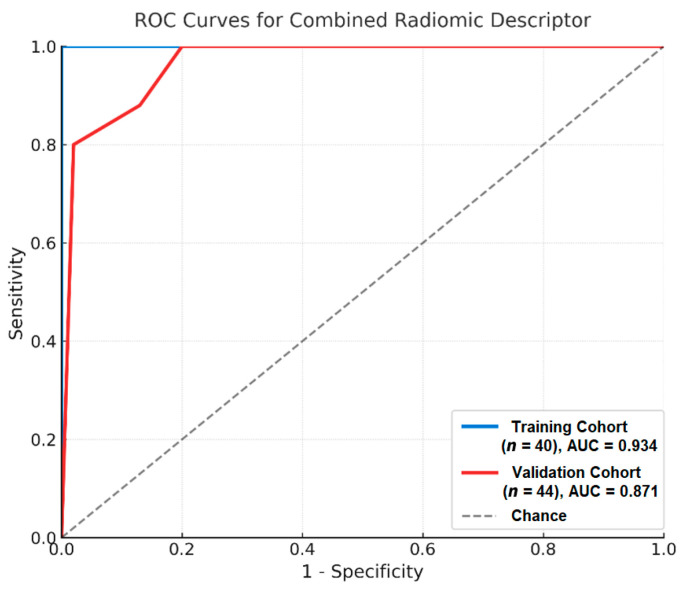
Receiver operating characteristic (ROC) curves for the training (blue) and prospective internal validation (red) cohorts demonstrating the diagnostic performance of the combined radiomic descriptor for muscle-invasive bladder cancer. AUC (95% CI): training 0.934 (0.853–1.000); validation 0.871 (0.768–0.974). Thresholds were fixed from training and applied unchanged to validation.

The evaluation of the descriptor in the training cohort yielded a sensitivity of 100% and a specificity of 96.3%, with an AUC of 0.934. Application to the validation cohort (*n* = 44) resulted in a sensitivity of 85.7% and a specificity of 96.2%, with an AUC of 0.871 (Figure 3), a modest decrease expected with temporal splits—likely due to a slightly different case mix (higher T3–T4; Table 2), and no re-tuning of training-derived cut-offs; 95% CIs overlapped, indicating preserved discrimination. Both ROC models reached statistical significance (*p* < 0.05). The performance metrics for both cohorts are summarized in Table 4.

## 4. Discussion

Radiomics—a rapidly evolving field in oncologic imaging—enables the extraction of high-dimensional quantitative features from medical images, offering an objective assessment of tumor heterogeneity beyond conventional visual analysis [12,13]. The integration of machine learning and radiomic feature selection aims to develop classifiers that can provide clinically meaningful information for patient management, including tumor staging, prognostic assessment, and therapy response prediction [14,15]. Accurate preoperative evaluation of bladder cancer (BC) invasion depth is essential for determining optimal treatment strategies. Rather than claiming superiority, our goal was to demonstrate a compact, reproducible radiomics approach that may complement routine MRI assessment.

Although the introduction of the standardized VI-RADS system has improved the diagnostic performance of multiparametric MRI (mpMRI) for muscle-invasive bladder cancer (MIBC), limitations remain. Several studies report that even experienced readers achieve sensitivity and specificity of only 83.4% and 77.3%, respectively, while less experienced readers perform slightly worse (82.0% and 73.9%) [16]. This variability underscores the need for more objective, reproducible approaches that can reduce interobserver variability and improve diagnostic consistency across different clinical settings.

In this context, recent research has increasingly focused on radiomics analysis of preoperative MRI data as a means to enhance diagnostic performance in BC. For instance, Ma et al. (2022) and Xu et al. (2021) demonstrated that radiomics-based machine learning models using multiparametric MRI can accurately distinguish muscle-invasive (MIBC) from non-muscle-invasive bladder cancer (NMIBC), with reported AUC values typically ranging from 0.80 to 0.92 [15,17,18,19,20]. These models leverage quantitative texture features—such as gray-level co-occurrence and run-length matrices—to capture subtle differences in tissue heterogeneity that may not be apparent to the human eye. Notably, both 2D and 3D texture analysis approaches have been shown to provide high diagnostic sensitivity and specificity in differentiating tumor stages [21,22].

Beyond staging, the application of radiomics has expanded to predicting therapeutic response and patient outcomes. Several groups have demonstrated that radiomic signatures derived from baseline mpMRI or CT scans can serve as biomarkers for response to neoadjuvant chemotherapy or immunotherapy, often achieving robust accuracy metrics (AUC 0.75–0.87) [23,24,25]. Furthermore, prognostic modeling using radiomics has shown encouraging results: studies by Wang et al. (2022) and Feng et al. (2021) reported that specific radiomic profiles are independently associated with relapse-free survival and recurrence risk, both in training and external validation cohorts [26,27]. These findings illustrate that radiomics can serve as a versatile tool—not only for staging but also for guiding treatment planning and predicting long-term outcomes.

Further evidence supporting the clinical potential of radiomics comes from recent methodological advances. Ye et al. showed that the semi-automatic segmentation of bladder tumors on T2-weighted MRI achieves diagnostic performance comparable to manual segmentation (AUC ~0.89–1.00, *p* < 0.05), while significantly reducing analysis time, thus facilitating broader clinical implementation [28]. Similarly, Özdemir et al. (2024) found that combining standard VI-RADS scoring with radiomic features improved diagnostic accuracy for muscle invasion (AUC = 0.92 ± 0.12) compared to radiomics alone (AUC = 0.83 ± 0.22), highlighting the benefit of integrating radiomics into existing clinical frameworks [29]. Complementing these results, a recent review by Arita et al. (2025) emphasized the promise of artificial intelligence and quantitative imaging biomarkers—including mpMRI radiomics—for improving treatment response assessment and prognosis in bladder cancer and advocated for multicenter validation to further enhance clinical applicability [30].

Against this backdrop, our study contributes additional evidence by developing and validating a multiparametric, 3D, mpMRI-based radiomic descriptor for preoperative, noninvasive detection of muscle invasion in bladder cancer. Our approach achieved high sensitivity and specificity in both the training and prospective validation cohorts. We selected GLRLM-LRLGE (from T2) and GLRLM-SRLGE (from ADC) because they repeatedly ranked among the top univariate performers with balanced sensitivity and specificity and provided complementary information—with T2 reflecting morphologic contrast and ADC capturing diffusion-related microstructural heterogeneity—thereby limiting redundancy while keeping the model interpretable. Notably, second-order texture features from both T2-weighted and ADC sequences demonstrated strong discriminative ability, with sensitivity and specificity ranging from 70% to 95%, and area under the curve (AUC) values between 0.7 and 0.9. These results align with previous reports for both T2-weighted imaging [20,26] and DWI/ADC-derived features [25,26]. In our dataset, DWI features did not show statistically significant group differences, echoing recent findings that ADC-based models are often more robust than those using DWI alone [31]. Moreover, consistent with the literature, mpMRI models utilizing multiple sequences generally outperform those based on a single sequence [32,33]. In validation, a modest reduction in AUC is expected with temporal splits. It likely reflects a slightly different case mix and the absence of re-tuning (training-defined thresholds were locked), with preserved discrimination given the overlapping 95% CIs.

A notable strength of our methodology is the use of 3D volume-based, multivariate analysis, which aligns with current best practices in radiomic research and enhances reproducibility compared with 2D slice-based approaches [14]. Importantly, we constructed a simplified descriptor combining just two robust features (GLRLM-LRLGE from T2 and GLRLM-SRLGE from ADC), enabling accurate classification with minimal input. This model achieved a sensitivity of 85.7%, specificity of 96.2%, and AUC of 0.871 in the prospective internal validation cohort. In clinical workflow, the descriptor could be applied after a standard VI-RADS read to support decisions in borderline cases; the binary AND rule (both features above fixed cut-offs) and the small feature set facilitate rapid second-look without extending MRI scan time.

### 4.1. Clinical Implications

Our findings suggest that a compact radiomic descriptor derived from routine T2-weighted and ADC data can complement standard mpMRI reads in everyday practice, particularly for adjudicating borderline cases where management hinges on suspected muscle invasion.

### 4.2. Comparison with Clinical Standards (VI-RADS)

This work is not intended to replace VI-RADS but to augment it with objective, reader-independent measurements of heterogeneity. A head-to-head, multi-reader comparison between VI-RADS, the radiomic descriptor, and integrated models (VI-RADS + radiomics) is warranted to determine incremental value and define clinical roles.

### 4.3. Technical and Workflow Consideration

The descriptor uses only two scalar features with fixed, training-defined thresholds, yielding a minimal computational footprint and straightforward PACS/RIS integration after lesion segmentation. Semi-automatic segmentation—shown to approximate manual accuracy while reducing time—represents a pragmatic path to streamline adoption. Multi-vendor 1.5 T imaging under a harmonized VI-RADS-based protocol was used; nonetheless, cross-site calibration and feature harmonization should be addressed in future work.

### 4.4. Limitations

The sample size was relatively small, and all imaging was performed on 1.5 T MRI scanners, whereas some studies have used 3 T systems. The single-center design may have introduced selection bias, though the prospective validation cohort enhances the robustness of our findings. Additionally, only patients with urothelial carcinoma were included, and in multifocal cases, analysis was restricted to the largest lesion, which may limit generalizability. TUR-based histology may underestimate invasion due to sampling limitations, particularly in large or multifocal tumors. We partially mitigated this by incorporating cystectomy histology when available and using a narrow MRI-to-reference window; nevertheless, some misclassification bias is possible. By ensuring the presence of muscularis propria in all TURBT specimens and incorporating cystectomy histology when available, we strengthened the reliability of the reference standard and reduced the likelihood of under-staging. We acknowledge, however, that this design choice may limit generalizability to settings where TURBT specimens occasionally lack muscularis propria.

We did not perform internal cross-validation; instead, a temporal internal validation with locked training-defined thresholds was used to assess out-of-sample discrimination without re-tuning. While this design reduces information leakage, it may not fully capture model variability across resamples.

Imaging was acquired on two vendors (Toshiba Vantage Titan 1.5 T (Toshiba Medical Systems Corporation, Otawara, Japan) and a SIGNA Voyager 1.5 T (GE Healthcare, Waukesha, WI, USA)) under a harmonized VI-RADS–based protocol with identical preprocessing (absolute resampling; 128 gray levels per LifeX guidance). No formal cross-vendor harmonization was applied, so residual scanner-related effects on feature distributions cannot be excluded and should be addressed in multicenter studies.

Despite these limitations, our results support the growing body of evidence indicating that radiomics can provide objective, noninvasive tools for bladder cancer staging. Further validation in larger, multicenter prospective cohorts—including diverse MRI field strengths and patient populations—will be essential for clinical translation.

### 4.5. Future Directions

Future work will be concentrated on a prospective, multi-reader, head-to-head comparison between the compact radiomic descriptor and conventional MRI assessment (e.g., VI-RADS), together with evaluation of integrated models (VI-RADS + radiomics) to quantify incremental value, delineate clinical roles, and assess utility in routine workflows. Multicenter, multi-vendor validation (including 1.5 T and 3 T scanners), robustness testing (repeatability/reproducibility and segmentation variability), and reporting of calibration and clinical-impact metrics will be essential steps toward clinical translation.

In sum, diagnostic performance was high relative to the histologic reference in a single-center setting, and a compact two-feature descriptor preserved discrimination on prospective internal validation. These results support mpMRI-based radiomics as a decision-support adjunct to routine assessment—particularly in borderline cases—pending multicenter, multi-vendor validation and workflow studies.

## 5. Conclusions

Radiomics based on texture analysis of T2-weighted images and ADC demonstrated robust diagnostic performance for the preoperative identification of muscle invasion in bladder cancer relative to the histologic reference in a single-center, temporally validated setting. A compact two-feature descriptor (GLRLM-LRLGE from T2 and GLRLM-SRLGE from ADC) delivered excellent performance in the training cohort and strong discrimination in the prospective internal validation cohort while relying on fixed, training-defined thresholds. Taken together, these results indicate that combined T2/ADC radiomics can be integrated as a decision support tool alongside routine mpMRI (e.g., in borderline T1–T2 cases) and support clinical translation. To consolidate adoption and ensure generalizability, the next steps concern multicenter, multi-vendor external validation (1.5 T/3 T), repeatability/reproducibility testing (including segmentation robustness and, where feasible, test–retest), and workflow/impact evaluations (e.g., decision curve analysis and streamlined semi-automatic or automatic segmentation).

## Figures and Tables

**Table 1:** Overview of radiomic texture features extracted for analysis.

Feature Category	Number of Features	Features
First-order Statistics	6	Skewness, Kurtosis, Entropy_log10, Entropy_log2, Energy, AUC_CSH
Gray-Level Co-occurrence Matrix (GLCM)	6	Homogeneity, Energy, Contrast, Correlation, Entropy, Dissimilarity
Gray-Level Run Length Matrix (GLRLM)	11	SRE, LRE, LGRE, HGRE, SRLGE, SRHGE, LRLGE, LRHGE, GLNU, RLNU, RP
Neighborhood Gray-Level Difference Matrix (NGLDM)	3	Coarseness, Contrast, Busyness
Gray-Level Zone Length Matrix (GLZLM)	11	SZE, LZE, LGZE, HGZE, SZLGE, SZHGE, LZLGE, LZHGE, GLNU, ZLNU, ZP

Notes: SRE: short-run emphasis; LRE: long-run emphasis; LGRE: low gray-level run emphasis; HGRE: high gray-level run emphasis; SRLGE: short-run low gray-level emphasis; SRHGE: short-run high gray-level emphasis; LRLGE: long-run low gray-level emphasis; LRHGE: long-run high gray-level emphasis; GLNU: gray-level non-uniformity; RLNU: run length non-uniformity; RP: run percentage; SZE: short-zone emphasis; LZE: long-zone emphasis; LGZE: low gray-level zone emphasis; HGZE: high gray-level zone emphasis; SZLGE: short-zone low gray-level emphasis; SZHGE: short-zone high gray-level emphasis; LZLGE: long-zone low gray-level emphasis; LZHGE: long-zone high gray-level emphasis; ZLNU: zone length non-uniformity; ZP: zone percentage.

**Table 2:** Baseline demographic and clinical characteristics of patients in the training and validation cohorts.

Characteristic	Subcategory	Training Cohort (*n* = 40)	Validation Cohort (*n* = 44)	*p*-Value
Sex, n (%)	Male	31 (77.5%)	36 (81.8%)	0.62 (Pearson’s χ^2^)
	Female	9 (22.5%)	8 (18.2%)	
Age, years	Mean ± SD	64.5 ± 12.3	64.0 ± 9.7	0.92 (Mann–Whitney U-test)
	IQR	58–70	59–72	
Histology	Transitional cell carcinoma	40 (100%)	44 (100%)	–
Reference standard (histology source)	TURBT	31 (77.5%)	34 (77.27%)	0.54 (Pearson’s χ^2^)
	Cystectomy	9 (22.5%)	7 (15.91%)	
Muscle invasion, *n* (%)	Present	21 (52.5%)	28 (63.6%)	0.30 (Pearson’s χ^2^)
	Absent	19 (47.5%)	16 (36.4%)	
Maximum tumor dimension, mm	Mean ± SD	32 ± 16.5	40 ± 22.0	0.27 (Mann–Whitney U-test)
Tumor stage, *n* (%)	T1	21 (52.5%)	18 (40.9%)	0.14 (Pearson’s χ^2^ 4 × 2)
	T2	14 (35.0%)	11 (25.0%)	
	T3	3 (7.5%)	8 (18.2%)	
	T4	2 (5.0%)	7 (15.9%)	

Notes: Quantitative data are presented as mean ± standard deviation (SD) or IQR (interquartile range). Statistical comparisons between cohorts were performed using Pearson’s chi-square test for categorical variables and the Mann–Whitney U-test for continuous variables. All patients had transitional cell carcinoma. Maximum tumor dimension was measured on MRI using Radiant Dicom Reader. Muscularis propria was present in all TURBT specimens included (100%).

**Table 3:** Selected radiomic features with highest diagnostic performance in training cohort.

MRI Sequence	Feature Class	Feature Name	AUC (95% CI)	Sensitivity (%)	Specificity (%)
T2	First-order	AUC_CSH	0.701 (0.53–0.88)	72.2	75.0
	GLRLM	HGRE	0.736 (0.57–0.90)	88.9	75.0
	GLRLM	SRLGE	0.837 (0.70–0.98)	77.8	93.8
	GLRLM	SRHGE	0.736 (0.57–0.90)	88.9	75.0
	GLRLM	LRLGE	0.837 (0.70–0.98)	77.8	93.8
	GLRLM	LRHGE	0.740 (0.57–0.91)	83.3	75.0
	GLRLM	GLNU	0.847 (0.71–0.98)	72.2	93.8
	GLZLM	HGZE	0.914 (0.58–0.92)	94.4	75.0
	GLZLM	SZLGE	0.840 (0.70–0.98)	72.2	93.8
	GLZLM	SZHGE	0.726 (0.56–0.90)	94.4	68.8
	GLZLM	LZLGE	0.816 (0.67–0.96)	77.8	81.2
ADC	First-order	AUC_CSH	0.701 (0.53–0.88)	72.2	75.0
	First-order	RIM_min	0.906 (0.80–1.00)	72.2	100.0
	GLCM	Energy	0.708 (0.53–0.88)	55.6	87.5
	GLRLM	SRE	0.719 (0.54–0.89)	44.4	100.0
	GLRLM	HGRE	0.736 (0.57–0.90)	88.9	75.0
	GLRLM	SRLGE	0.837 (0.70–0.98)	77.8	93.8
	GLRLM	SRHGE	0.736 (0.57–0.90)	88.9	75.0
	GLRLM	LRLGE	0.837 (0.70–0.98)	77.8	93.8
	GLRLM	LRHGE	0.740 (0.57–0.91)	83.3	75.0
	GLRLM	GLNU	0.847 (0.71–0.98)	72.2	93.8
	GLZLM	HGZE	0.884 (0.58–0.91)	94.4	75.0
	GLZLM	SZLGE	0.840 (0.70–0.98)	72.2	93.8
	GLZLM	SZHGE	0.726 (0.56–0.90)	94.4	68.8
	GLZLM	LZLGE	0.816 (0.67–0.96)	77.8	81.2
	GLZLM	LZHGE	0.701 (0.53–0.88)	100.0	37.5

Notes: AUC—area under the ROC curve; CI—confidence interval; ADC—apparent diffusion coefficient; GLCM—gray-level co-occurrence matrix; GLRLM—gray-level run length matrix; GLZLM—gray-level zone length matrix. Sensitivity and specificity are shown for the optimal cut-off (Youden index).

**Table 4:** Diagnostic accuracy of the combined radiomic descriptor in training and validation cohorts.

Metric	Training Cohort (*n* = 40)	Validation Cohort (*n* = 44)
Pearson’s correlation coefficient (r)	0.338	0.237
*p*-value	0.040	0.012
Sensitivity (%)	100.0	85.7
Specificity (%)	96.3	96.2
AUC (mean ± SD)	0.934 ± 0.084	0.871 ± 0.121
AUC (95% CI)	[0.853–1.000]	[0.768–0.974]

Notes: AUC—area under the ROC curve; SD—standard deviation. The combined descriptor is considered positive if both the GLRLM-LRLGE_T2 and GLRLM-SRLGE_ADC values exceed their respective cut-off thresholds. Statistical significance was set at *p* < 0.05. CIs computed using the Hanley–McNeil variance.

## Data Availability

The original contributions presented in this study are included in the article. Further inquiries can be directed to the corresponding author.

## Abbreviations

The following abbreviations are used in this manuscript:

ADC	Apparent Diffusion Coefficient
AI	Artificial Intelligence
AUC	Area Under the Curve
BC	Bladder Cancer
BCG	Bacille Calmette–Guerin
CI	Confidence Interval
DWI	Diffusion-Weighted Imaging
GLCM	Gray-Level Co-occurrence Matrix
GLRLM	Gray-Level Run Length Matrix
LRLGE	Long-Run Low Gray-Level Emphasis
MD	Median
MIBC	Muscle-Invasive Bladder Cancer
mpMRI	Multiparametric Magnetic Resonance Imaging
MRI	Magnetic Resonance Imaging
NMIBC	Non-Muscle-Invasive Bladder Cancer
*p*	*p*-value (probability value)
ROC	Receiver Operating Characteristic
SD	Standard Deviation
SEM	Standard Error of the Mean
SRLGE	Short-Run Low Gray-Level Emphasis
TUR	Transurethral Resection
VI-RADS	Vesical Imaging Reporting and Data System
VOI	Volume of Interest
